# Carbon Nanotubes Enhance Cytotoxicity Mediated by Human Lymphocytes *In Vitro*

**DOI:** 10.1371/journal.pone.0021073

**Published:** 2011-06-22

**Authors:** Zhao Sun, Zhe Liu, Jie Meng, Jie Meng, Jinhong Duan, Sishen Xie, Xin Lu, Zhaohui Zhu, Chen Wang, Shuchang Chen, Haiyan Xu, Xian-Da Yang

**Affiliations:** 1 Peking Union Medical College Hospital, Chinese Academy of Medical Sciences & Peking Union Medical College, Beijing, China; 2 Institute of Basic Medical Sciences, Chinese Academy of Medical Sciences & Peking Union Medical College, Beijing, China; 3 National Center of Nanoscience and Technology, Beijing, China; 4 Institute of Physics, Chinese Academy of Sciences, Beijing, China; Aristotle University of Thessaloniki, Greece

## Abstract

With the expansion of the potential applications of carbon nanotubes (CNT) in biomedical fields, the toxicity and biocompatibility of CNT have become issues of growing concern. Since the immune system often mediates tissue damage during pathogenesis, it is important to explore whether CNT can trigger cytotoxicity through affecting the immune functions. In the current study, we evaluated the influence of CNT on the cytotoxicity mediated by human lymphocytes *in vitro*. The results showed that while CNT at low concentrations (0.001 to 0.1 µg/ml) did not cause obvious cell death or apoptosis directly, it enhanced lymphocyte-mediated cytotoxicity against multiple human cell lines. In addition, CNT increased the secretion of IFN-γ and TNF-α by the lymphocytes. CNT also upregulated the NF-κB expression in lymphocytes, and the blockage of the NF-κB pathway reduced the lymphocyte-mediated cytotoxicity triggered by CNT. These results suggest that CNT at lower concentrations may prospectively initiate an indirect cytotoxicity through affecting the function of lymphocytes.

## Introduction

Carbon nanotubes (CNTs) have been shown to have potential applications in multiple biomedical fields[1], [2], [3], [4], [5], [6], [7], [8], [9], [10], especially as effective transporters for delivery of various bioactive molecules such as peptides[11], proteins[12], [13], [14], DNAs[15], [16], [17], RNAs[18], or drugs[19], [20]. Our previous study revealed that CNT conjugated tumor protein could enhance the uptake of tumor antigen by human dendritic cell (DC) and the capability of DC to induce anticancer response in vitro. The results suggest that CNT-based nanotechnology may have a prospective role in the development of more efficacious DC-based anticancer immunotherapy.[21] With the expansion of the scope of prospective CNT applications, the toxicity of CNT to mammalian cells becomes an issue of great concern. Some literatures reported that exposing cells to CNT led to cell death[22], [23], apoptosis[24], or inhibition of proliferation[25], [26], while others showed that CNT at lower concentrations had minimal toxicity[27], [28], [29]and did not significantly affect the function and viability of cells[27]. So far however, most studies on CNT toxicity have focused on the changes in cells following direct CNT exposure. The indirect toxicity mediated by the immune modulation effects of CNT has not been well explored. Because the immune system often plays a major role in tissue damage during pathogenesis[30], in addition to studying the direct CNT toxicity on cells, it is also important to evaluate whether CNT can trigger cytotoxicity through affecting the function of lymphocytes. Here in this study, the effects of CNT on lymphocyte-mediated cytotoxicity on multiple human cell lines were assessed *in vitro*. Moreover, we evaluated whether CNT would influence the proliferation of lymphocytes, the production of IFN-γ and TNF-α by lymphocyte, and the activation of NF-κB in an attempt to explore the mechanisms of the prospective CNT-induced immune cytotoxicity.

## Results

### 1. Characterization of CNT

Functionalized CNT used in this study was prepared using a method similar to that described in our prior report[31]. Briefly, an oxidation/sonication procedure was utilized to introduce carboxyl groups to CNT surface for solubilization enhancement. The characterization of the functionalized CNT was carried out with standard methodology[32], [33]. Scanning electronic microscopy revealed that the tube-like structure of CNT was well maintained, with an average length of 500–800 nm and an average diameter of 20–30 nm. X-ray photoelectron spectroscopy analysis detected carboxyl groups on the surface of CNT. The resultant CNT solution had a concentration of 0.2 mg/ml (data not shown, the results have been describled in the paper we published before).[31]

### 2. CNT promoted lymphocyte-mediated cytotoxicity

**Figure 1:**
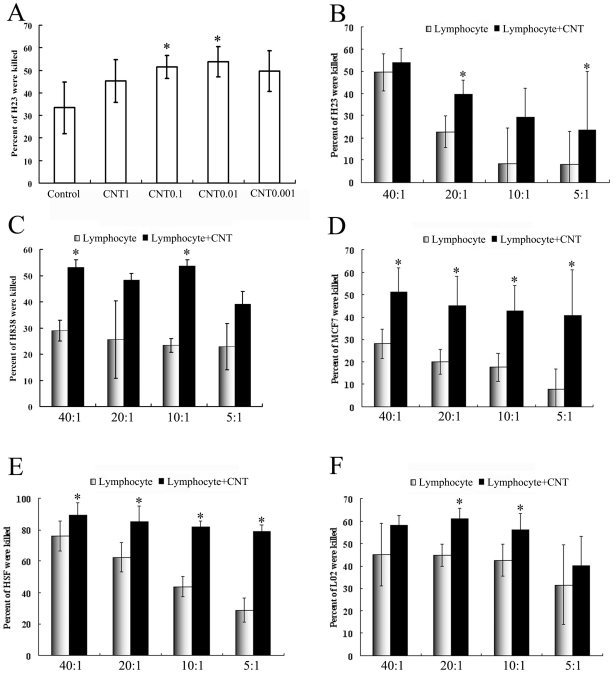
CNT promoted lymphocyte-mediated cytotoxicity. (A) Percentage of lived H23 cells was killed by Lymphocyte alone or co-incubation with CNT of various concentrations (n  =  6, ± SD). The star indicates a statistically significant difference between this group and the Lymphocyte alone groups (p<0.05). (B–F) Percentage of other cell lines (B: H23; C: H838; D: MCF7; E: HSF; F: L02) were killed by Lymphocyte alone or co-incubation with 0.01 µg/ml CNT on difference E: T ratios (n  =  6, ± SD). The star indicates a statistically significant difference between this group and the Lymphocyte alone groups (p<0.05).

The *in vitro* cytotoxiciy mediated by the lymphocytes were measured using a standard methodology that had been employed in *ex vivo* immune studies[34], [35]. To explore the effective working concentration of CNT, various concentrations of CNT (from 0.001 to 1 µg/ml) were tested, with the H23 cell line as the target cells and the effector to target cell raito (E: T ratio) of 10∶1. After incubation with various concentrations of CNT, the lymphocytes (effector cells) were thoroughly washed, and co-incubated with the target cells for 3 more days. The percentage of viable target cells was then evaluated with an MTS assay[34], [35]. The results showed that the effective working concentration of CNT was between 0.01 to 0.1 µg/ml, with the growth inhibition rates of 53.84±6.77% and 51.53±5.17%, respectively (Fig.1A, p<0.05, compare to control group). The control group used lymphocytes that had not been exposed to CNT, and generated a growth inhibition rate of 33.41±11.44%. The CNT groups of 1 µg/ml and 0.001 µg/ml did not enhance the growth inhibition to a statistically significant level compared to the control.

To confirm the immune modulatory effect of CNT, we next tested whether CNT of 0.01 µg/ml would also induce a lymphocyte-mediated cytotoxicity against other human cell lines, across a wider range of E: T ratios (from 40∶1 to 5∶1). As shown in Fig 1, CNT of 0.01 µg/ml induced cell growth inhibition consistently across various human cell lines, including three tumor cell lines (MCF7, H838 and H23) and two non-tumor cell lines (HSF and L02). When the MCF7 was used as the target cells, the CNT induced growth inhibitions were 51.23±10.72%, 45.08±12.58%, 42.65±11.3% and 40.73±20.26%, for E/T ratios of 40∶1, 20∶1, 10∶1 and 5∶1, respectively (p<0.05, compared to control); whereas the control group generated much lower inhibitions of 28.15±6.42%, 20.1±5.63%, 17.66±6.21% and 8.08±9.01% respectively. Similar trends were observed in experiments with other cell lines (Fig 1), suggesting that a low concentration of CNT could indeed enhance the lymphocyte-mediated cytotoxicity *in vitro.*

### 3. CNT did not influence cell Viability

**Figure 2:**
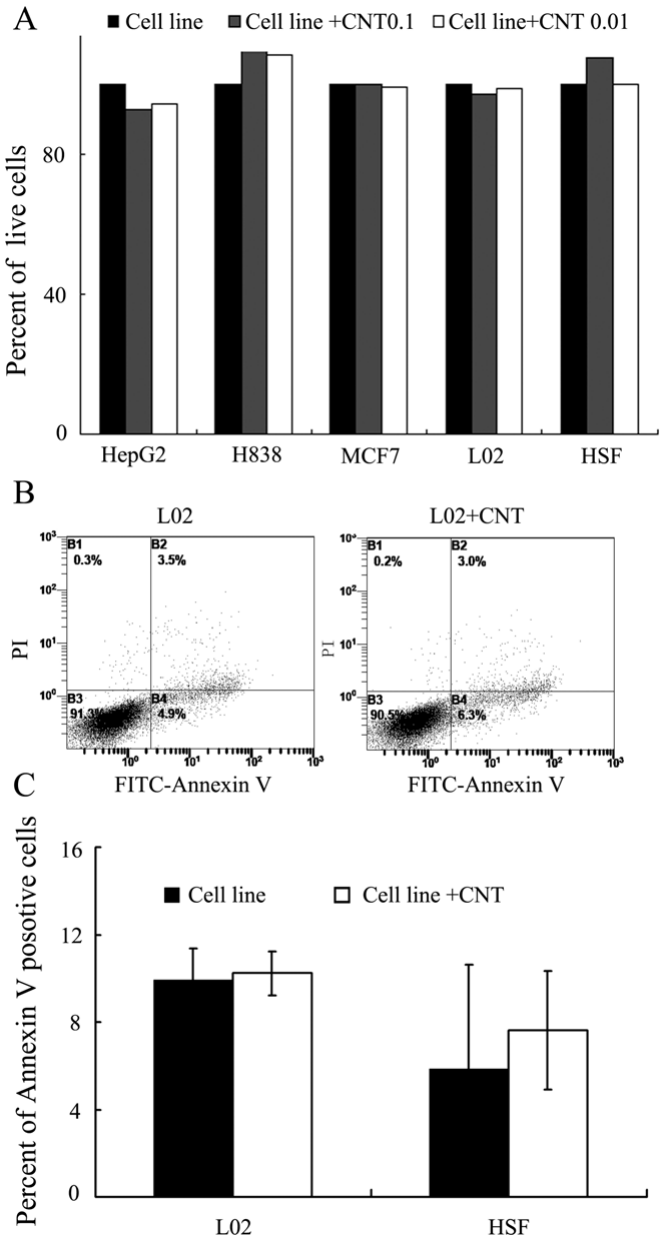
Influence of CNT on cell Viability. (A) Percentage of different cell lines after co-incubation with CNT of various concentrations (n  =  6, ± SD). (B, C) Effect of CNT on L02 cell apoptosis. The test was conducted by Annexin-V and PI double staining and analyzed by flow cytometry. Apoptosis of L02 cells was analyzed in L02 cells alone (L02), CNT co-cultured with L02 (L02+CNT), Annexin V + means the cells were PI negative and Annexin V positive. Data are shown as means ± SD of three independent experiments.

It is important to investigate whether a prospective CNT toxicity on the target cells was responsible for the higher tumor inhibition observed in Fig. 1. To address this issue, the target cells were incubated for 3 days in either normal medium or mediums containing CNT of 0.01 or 0.1 µg/ml. The viability of the MCF7 cells was then evaluated by the MTS assay. The results showed no significant difference in the number of live cells among the groups (Fig 2A) further explore the issue, we also tested whether CNT of 0.01 µg/ml would induce an apoptosis effect in L02 and HSF cell lines. As shown in Fig 2B, C CNT failed to induce an obvious apoptosis in either cell lines at this concentration. These results suggested that the direct cytotoxicity of CNT was not the major mechanism of the enhanced growth inhibition observed in Fig 1.

### 4. Influence of CNTs on lymphocyte proliferation

**Figure 3:**
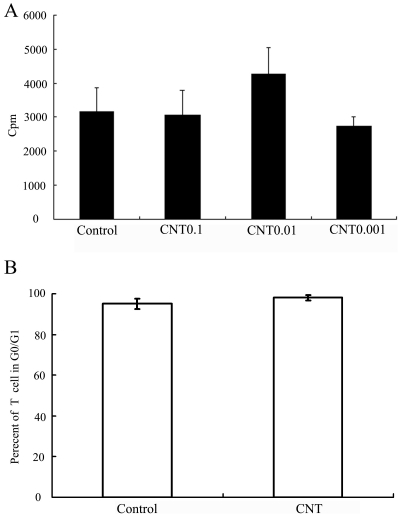
Influence of CNT on lymphocyte proliferation. (A) Influence of CNT on lymphocyte proliferation, lymphocytes were cultured alone or co-cultured with CNT of various concentrations for 3 days. [3 H]-thymidine were added at the last 18 hour. (B) Influence of CNT on cell cycle of lymphocyte. Lymphocytes were cultured alone or co-cultured with CNT for 3 days, then the cell were collected and detected by PI.

To explore the mechanism by which CNT enhanced lymphocytes' cytotoxicity, we next evaluated the influence of CNT on lymphocyte proliferation. CNT of various concentrations (0.001, 0.01, and 0.1 µm/ml) were added to the culture medium, and lymphocyte proliferation was subsequently evaluated with the standard technique of [3 H]-thymidine uptake. The results showed no significant difference among the groups, though CNT of 0.01 µm/ml tended to raise the uptake of [3 H]-thymidine. (Fig 3A) Moreover, there was no difference in the percentage of lymphocytes that stayed in G0/G1 phases (Fig 3B).

**Figure 4:**
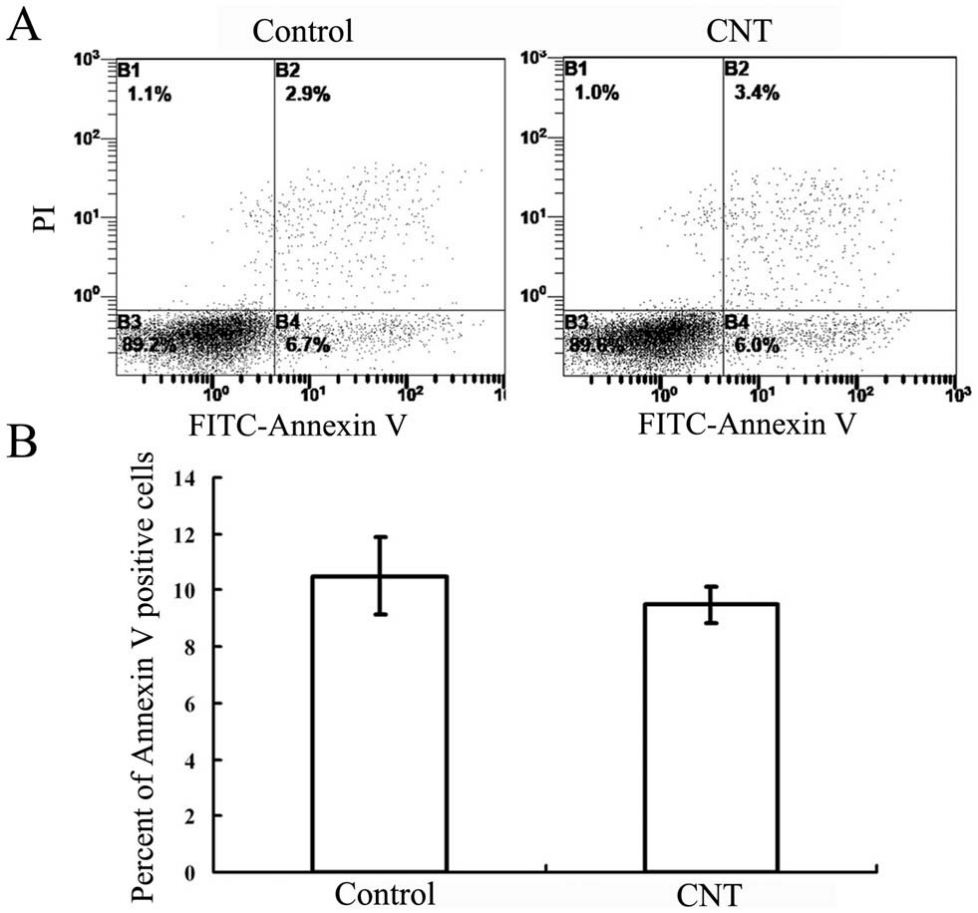
Influence of CNT on lymphocyte apoptosis. The test was conducted by Annexin-V and PI double staining and analyzed by flow cytometry. Apoptosis of lymphocyte cells was analyzed in lymphocyte cells alone (Lymphocyte), CNT co-cultured with lymphocyte (Lymphocyte+CNT), Annexin V + means the cells were PI negative and Annexin V positive. Data are shown as means ± SD of five independent experiments.

We also investigated whether CNT would promote the apoptosis of the lymphocytes. As shown in Fig 4, CNT did not induce an obvious apoptosis in lymphocytes either. These results showed that CNT did not significantly affect the lymphocytes proliferation or apoptosis, suggesting that the enhanced lymphocyte-mediated cytotoxicity was probably mediated through other mechanisms.

**Figure 5:**
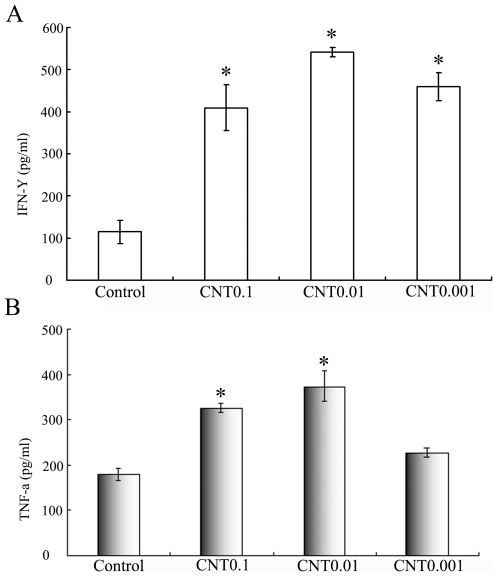
CNT promote lymphocytes secretion cytokines. Lymphocyte cell were cultured alone or co-cultured with CNT of various concentrations for 24 hours. The levels of secreted cytokines IFN-γ and TNF-α were measured in culture supernatants by ELISA.

We next evaluated if CNT would affect the secretion of proinflammatory cytokines IFN-γ and TNF-α, which are generally regarded as the key signs of lymphocyte activation. Lymphocytes were cultured in medium containing CNT of either 0.001, 0.01, or 0.1 µg/ml for 24 hours. The levels of secreted proinflammatory cytokines IFN-γ and TNF-α were measured in culture supernatants using a standard double-sandwich ELISA protocol. As presented in Fig 5, lymphocyte cultured with CNT of 0.01 µg/ml or 0.1 µg/ml produced significant higher amounts of both cytokines compared with the untreated cells (p<0.05). The results suggested that CNT at proper concentrations might promote lymphocyte activation and the secretion of proinflammatory cytokines.

### 5. CNT promote lymphocyte cytotoxicity by NF-κB

**Figure 6:**
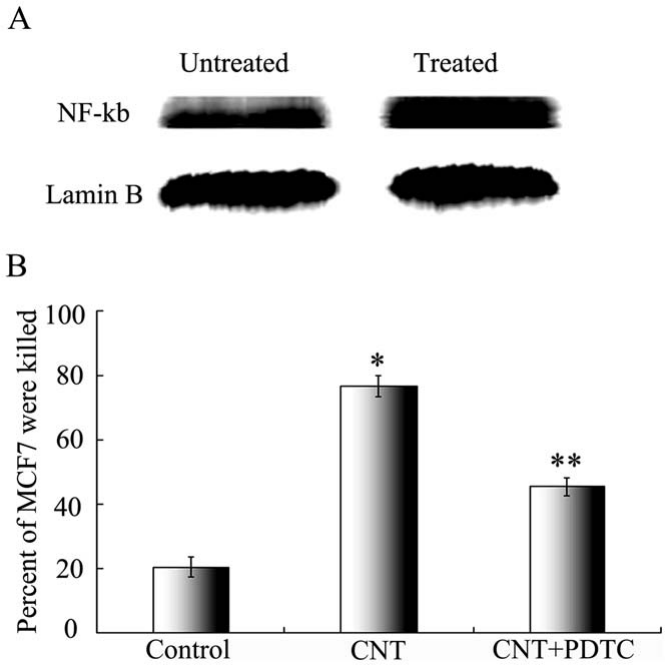
CNT promote lymphocytes Cytotoxicity by NF-κB. (A) The influence of CNT on the concentration of NF-κB p65 in the lymphocyte cell nuclei. Lymphocytes were incubated in either normal medium or that contains CNT of 0.01 µg/ml for 24 hours. Nuclear protein was extracted and the western blotting was utilized to compare the NF-κB activation. Lamin B was used as control. (B) The influence of NF-κB inhibitor PDTC on the lymphocyte-mediated cytotoxicity of CNT. MCF7 were co-cultured with Lymphocyte or Lymphocyte added CNT, or Lymphocyte added CNT and PDTC. The percent of lived MCF7 cell were detected by MTS.

To further explore the mechanism of CNT induced lymphocyte activation, we evaluated the influence of CNT on the NF-κB pathway in lymphocyte, which is a major transcription factor that regulates genes responsible for both the innate and adaptive immune responses. Lymphocytes were incubated in either normal medium or that contains CNT of 0.01 µg/ml. Nuclear protein was extracted and the western blotting was utilized to compare the NF-κB activation in the two groups of lymphocytes. The results showed that the NF-κB was more activated in the CNT-treated group (Fig. 6A), Lamin B was used as a control. To further explore the role of NF-κB activation in CNT induced cytotoxicity, the effects of NF-κB inhibitor PDTC was evaluated during cytotoxicity experiments. As presented in Fig 6B, while CNT enhanced the cytotoxicity against MCF7 cells by the lymphocytes, PDTC decreased the toxic effect significantly (p<0.05 compared to the CNT group). These results suggested that the activation of NF-κB probably involved in the lymphocyte-mediated toxicity induced by CNT exposure.

## Discussion

The toxicity and biocompatibility of CNT have become issues of great concern with the growth of prospective applications of CNT in biomedical fields. The aim of this study was to evaluate the influence of CNT on lymphocyte-mediated cytotoxicity *in vitro*, since the immune system often mediates tissue damage during pathogenesis. The results showed that CNT at low concentration (0.001 to 0.1 µg/ml) enhanced the immune-mediated cytotoxicity against multiple types of human cells *in vitro* (Fig 1), but did not cause obvious cell death or apoptosis directly (Fig 2). In addition, CNT increased the secretion of cytokines signaling the activation of lymphocytes, including -γ and TNF-α (Fig 5), but failed to trigger a proliferation of the lymphocytes (Fig 4). Furthermore, CNT upregulated the NF-κB expression in immune cells, and the blockage of the NF-κB pathway reduced the CNT-induced cytotoxicity by lymphocytes (Fig 6). These results suggest that CNT at lower concentrations may trigger changes in lymphocytes, which in turn may cause an indirect cytotoxicity.

Dumorter et al reported that CNTs could be uptaken by lymphocytes and macrophages *in vitro* without affecting the cell viability, and that CNT did not influence the functional activity of the immune cells[27]. They also noticed that CNT provoked the secretion of proinflammatory cytokines by macrophages. In agreement with their findings, the results of this study showed that CNT could enhance the secretion of IFN-γ and TNF-α by lymphocytes/PBMC. In addition, we observed that CNT also increased the lymphocyte-mediated cytotoxicity against multiple human cell lines *in vitro*. The enhanced cytokine secretion by the lymphocytes could be partially responsible for the enhanced immune cytotoxicity induced by CNT, since IFN-γ and TNF-α both mediate inflammatory reactions.

NF-κB is a major transcription factor that regulates genes responsible for both the innate and adaptive immune response, including those involved in lymphocyte development, maturation and proliferation. It has been reported that CNT could activate the NF-κB pathways in a few types non-immune cells, including keratinocytes[25], mesothelial cells[22], and lung cancer A549 cells[23]. However, it was unknown whether CNT could also activate the NF-κB pathway in lymphocytes. Here we observed for the first time that CNT could upregulate NF-κB expression in lymphocytes, and that blocking NF-κB reduced the CNT-induced immune cytotoxicity. The findings suggested that NF-κB was probably involved in the CNT-induced and lymphocyte-mediated cytotoxicity. However, it should be noted that cellular pathways other than NF-κB could not be excluded, and further studies are necessary to completely understand the transcriptional mechanism of the effects triggered by CNT.

The *in vivo* biological properties of CNT have also been evaluated in animal models in recent years. While CNT administered via the respiratory tract can cause severe lung damages[36], [37], [38], CNT given intravenously did not affect the long-term survival of the mice[39], [40], [41]. Prior research on the cellular toxicity of CNT mainly focused on the apoptosis or death of target cells following direct CNT exposure *in vitro*, while the indirect CNT toxicity mediated by the immune cells has not been reported in literature. Here we observed that CNT at low concentration (0.01 to 0.1 µg/ml) enhanced the lymphocyte-mediated cytotoxicity against multiple human cell lines *in vitro* (Fig 2), but failed to cause obvious cell death or apoptosis directly. Although this effect needs further evaluation with *in vivo* studies, the finding suggested yet another potential mechanism for CNT induced-toxicity.

In summary, while low-dose CNT failed to induce apoptosis in target cells directly, they can enhance the lymphocyte-mediated cytotoxicity *in vitro*. The results suggest that CNT may potentially trigger an indirect cytotoxicity through enhancing the function of lymphocytes.

## Materials and Methods

### 1. Preparation and Characterization of Functionalized CNT

Multiwalled carbon nanotubes were purchased from Chengdu Organic Chemicals Co. Ltd., with the purity of greater than 95%, diameter of 20 to 30 nm, average length of 50 µm, amorphous carbon of less than 3%, ash (catalyst residue) of less than 1.5%, special surface area of greater than 233 m^2^/g, and the thermal conductivity of about 2000 W/m^.^k. Stable aqueous suspensions of purified and shortened CNT were prepared by oxidation and sonication of the purchased commercial product using a method described in our previous study[31]. In brief, CNT were suspended in a 3∶1 mixture of concentrated H_2_SO_4_/HNO_3_ and sonicated at 540 W for 45 s. The resulting mixture was filtered through a polycarbonate filter membrane of 2 µm pore (Millipore) and rinsed thoroughly till neutralized. The obtained CNT were dried completely and suspended in pure water at the concentration of 0.3 mg/ml by sonication. Centrifugation (5000 rpm for 30 min) removed the unreacted components from the solution to afford a stable suspension of CNT. The size and shape of CNT were evaluated by scanning electron microscopy. The surface element chemistry of the oxidized CNT was analyzed by X-ray photoelectric spectroscopy (XPS, VG Escalab MK II, UK). The CNT suspension was sonicated again prior to mixing with culture or reaction mediums for further application.

### 2. Cell Culturing

The human cell lines NCI-H23 (human lung adenocarcinoma), NCI-H838 (human lung adenocarcinoma), MCF-7 (human breast adenocarcinoma), L02(human hepatocyte) and HSF(human skin fibroblast) ,HepG2(human hepatic carcinoma) were maintained in RPMI 1640 medium (Gibco Life Technologies) supplemented with 10% fetal calf serum (FCS; Gibco), 100 U/mL penicillin, 100 µg/mL streptomycin in 5% CO_2_ and humidified atmosphere at 37°C. All cell lines were purchased from Cell Resource Center, IBMS, CAMS/PUMC).

### 3. Preparation of human peripheral blood mononuclear cells

Healthy volunteers' blood were collected as we previously described.[21] Peripheral blood mononuclear cells (PBMC) were obtained by Ficoll-Paque density gradient centrifugation. All donors were required to sign an informed consent form according to procedures approved by the Ethics Committee at Chinese Academy of Medical Sciences and Peking Union Medical College. The PBMC were suspended in RPMI 1640 medium supplemented with 10% (vol/vol) FCS, 2 mM L-glutamine, 0.1 mM nonessential amino acids (Life Technologies, Grand Island, NY), 1 mM sodium pyruvate, 100 U/mL penicillin, 100 µg/mL streptomycin, 1% HEPES buffer, and 10 µM 2-mercaptoethanol. The PBMC, consisting of mainly lymphocytes[35], were used as effector cells in the cytotoxicity studies.

### 4. Cytotoxicity Studies

The cytotoxicity experiments were conducted using the standard methods for *ex vivo* immune studies[35]. Specifically, the MTS cell viability assay was utilized to evaluate the condition of various target cells. MTS assay is a currently well-adopted method that is superior than the MTT assay in both sensitivity and applicability[34], [35]. The MCF7, H23, H838, HSF and L02 cells were employed here as the target cells. For various experimental groups the lymphocytes/PBMC were first incubated for 3 days with either normal RPMI 1640 medium, or mediums containing 0.1, 0.01 and 0.001 µg/ml of CNT respectively. After 3 days, the lymphocytes (effector cells) were removed and washed for 4 times with PBS, before mixing with the target cells for the cytotoxicity study.

The cytotoxicity study was performed in 96-well U-bottom plates according to standard protocols [35]. The target cells were added to the wells with culture medium and incubated for 4 hours so that the cells became adherent. Effector cells were mixed in the wells according to the indicated effector:target (E:T) ratios. The total volume per well was adjusted to 200 µl. The plate was then incubated in a CO_2_ incubator at 37°C for 3 days. The positive control and the background control were set up according to the instruction of the manufacturer (MTS Cell Titer Kit, Promega) and each used 6 wells. The background control was taken from 6 wells containing medium and MTS solution. The positive control was taken from 6 wells containing the target cells, medium and MTS, but without the effector cells. After 3 days, the supernatants were removed, and the plates were washed thrice with PBS. One hundred µl RPMI 1640 and 20 µl MTS solution were added per well. The plates were incubated for about 90 minutes at 37°C. When the color turned to brown, the plates were measured for light absorption by an ELISA plate reader at 490 nm. Each experiment was repeated in 6 wells to ensure reliable readings. Results were compared by analysis of variance (ANOVA), using the SPSS13.0 software. P value of <0.05 was considered significant. The percentage cytotoxicity was calculated according to the following equation (A_490_ indicates the light absorption at 490 nm)[35]:
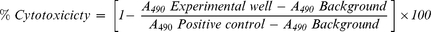



### 5. Cell Viability Assays after CNT Treatment

MCF7, H838, H23, L02, or HSF cells were washed twice with aseptic PBS, followed by a 10-minute centrifugation at 1000 rpm. The spun-down cells were re-suspended in RPMI 1640 culture medium supplemented with 10% FBS. Proper amount of CNT (0.1 µg or 0.01 µg) was added to 500 µl RPMI and thoroughly mixed, and incubated with 1×10^6^ cells at 37°C with 5% CO_2_ for 72 hours. The viability of the cells was then evaluated by the MTS method described above.

### 6. Effects of CNT on cell apoptosis

CNT and human cell lines were prepared as described above. Human cell lines L02 and HSF were cultured in either normal medium or that containing CNT (0.01 µg/ml) for 3 days. The cells were harvested and quantified, stained with Annexin-V kit (BD, USA), and analyzed with flow cytometry (FACS Vantage).

### 7. Effects of CNTs on lymphocyte apoptosis

CNT and lymphocytes were prepared as described above. Lymphocytes were cultured in either normal medium or that containing CNT (0.01 ug/ml) for 3 days. The cells were harvested and quantified, stained with Annexin-V kit (BD, USA), and analyzed with flow cytometry (FACS Vantage).

### 8. Effects of CNT on lymphocyte proliferation

The lymphocytes were incubated in RPMI 1640 medium with 10%FBS that contained CNT of 0.1, 0.01, or 0.001 µ g/ml for 72 hours. Control wells contained lymphocytes cultured in medium without CNT. Cultures were pulsed with 1 µCi/well [3 H]-TdR (Shanghai Nucleus Research Institute, China) on day 2, and harvested 18 hours later with a Tomtec automated harvester (Wallac Inc.). Thymidine uptake by lymphocytes was quantified with a liquid scintillation & luminescence counter (Wallac MicroBeta® TriLux).

### 9. Effects of CNT on cell cycle phase of lymphocytes

Lymphocytes were cultured alone or co-cultured with CNT for 3 days, then harvested and quantified. One million Lymphocytes were fixed with 70% cold ethanol at 4°C for 60 min, washed with PBS twice, and stained with 50 µg/ml PI (Sigma) at room temperature for 5 min. Flow cytometry of the lymphocytes was then performed. Data were analyzed with ModFIT software

### 10. Effects of CNT on IFN-γ and TNF-α Production

The lymphocytes were incubated in RPMI 1640 medium with 10%FBS that contained CNT of 0.1, 0.01, or 0.001 µg/ml for 72 hours. Control wells contained lymphocytes cultured in medium without CNT. Culture supernatants were collected. The IFN-γ and TNF-α in the supernatants were measured by standard sandwich ELISA techniques using the Quantikine Immunoassay Kit (Jingmei Inc, Shenzhen, China) according to the manufacturer's instructions. The plates were measured for light absorption by an ELISA plate reader (Bio-Rad) with a 450 nm filter.

### 11. Western blot assay of NF-κB

The lymphocytes were incubated in RPMI 1640 medium with 10%FBS and CNT of 0.01 µg/ml for 24 hours. Control wells contained lymphocytes cultured in medium without CNT. The nuclear proteins were extracted using a nuclear protein extraction kit (Nanjing KeyGen Biotech, China). Standard western blotting procedures were carried out with specific NF-κB p65 antibody (Santa Cruz), Lamin B antibody (Santa Cruz), and secondary antibodies labeled with horseradish peroxidase (Santa Cruz). Antibody/antigen complexes were detected using the ECL reagent (Millipore).

### 12. Inhibition effect of NF-κB by PDTC

To further explore the role of NF-κB activation in CNT induced cytotoxicity, the effects of NF-κB inhibitor PDTC (Pyrrolidinedithiocarbamic acid, ammonium salt) was evaluated during cytotoxicity experiments. As presented in Fig. 6, while CNT enhanced the cytotoxicity against MCF7 cells by the lymphocytes, PDTC decreased the toxic effect significantly (p<0.05 compared to the CNT group). These results suggested that the activation of NF-κB probably involved in the lymphocyte-mediated toxicity induced by CNT exposure.

### 13. Statistics

Statistical analysis was performed with the statistical SPSS 13.0 software. The nonparametric test was used to calculate the probability of significant differences among the groups. Statistical significance was defined as p <0.05.
